# Cross-sectional study in an out-of-hours primary care centre in northwestern Germany – patient characteristics and the urgency of their treatment

**DOI:** 10.1186/s12875-019-0929-4

**Published:** 2019-03-05

**Authors:** Insa Seeger, Laura Kreienmeyer, Falk Hoffmann, Michael H. Freitag

**Affiliations:** Department for Health Services Research, School of Medicine and Health Sciences, Carl von Ossietzky University Oldenburg, D-26111 Oldenburg, Germany

**Keywords:** Out-of-hours primary care (OOH primary care), Reason for encounter (RFE), Non-urgent, Physicians´ perspective

## Abstract

**Background:**

Due to the increasing number of non-urgent visits to emergency departments, it is becoming increasingly important to also investigate emergency care in out-of-hours (OOH) primary care. The aim of this study was to provide an insight into the care structures of an OOH primary care centre, to evaluate the reasons for encounter (RFE) and to assess the urgency of the treatment from the physicians´ point of view.

**Methods:**

In the summer of 2017, we conducted a cross-sectional study over four weeks in the OOH primary care centre of Oldenburg, a city in Lower Saxony with about 160,000 inhabitants. We collected socio-demographic data, RFE and the duration of the complaints. The International Classification for Primary Care 2nd Edition (ICPC-2) was used to categorize symptoms. The attending physicians supplemented information on further treatment (including hospitalization) and the urgency of consultation in the OOH primary care centre.

**Results:**

A total of 892 of the 1098 OOH patients which were visiting the OOH primary care centre took part in the study (participation: 81.2%). More than half of the patients were between 18 and 39 years old. A quarter of all RFE named by study participants were in the ICPC-2 category “skin”. More than 60% of patients had the symptoms for more than two days before visiting the OOH primary care centre. In 34.5% of all cases no medication was prescribed and one in six patients received further diagnostic tests such as urinalysis and blood tests (15.8%). From the physicians’ point of view, 26.3% of all study participants could have been treated by the family doctor during the regular consultation hours.

**Conclusion:**

The study shows that in the OOH primary care centre about a quarter of all patients could have waited until regular consultation hours. Mostly young patients used the easily accessible and free care in the OOH primary care centre. Further studies are necessary to better understand the individual reasons of patients to use the OOH primary care centre.

## Background

The increasing number of visits to the emergency department (ED) is an important issue in OECD countries and a significant proportion of ED visits are considered inappropriate, i.e. not necessary [1–4]. Non-urgent visits to EDs account for nearly 12% of ED visits in the United States, 20% in Italy, 25% in Canada, 31% in Portugal and 56% in Belgium [5]. The increasing number of non-urgent visits to the ED can also be noted in Germany. According to a survey of the German society for interdisciplinary emergency and acute medicine (DGINA) one third of all patients in the surveyed EDs could have been treated by general practitioners (GP) [6]. Such inappropriate emergency hospital visits could better be treated by GPs and specialists in outpatient practices or in out-of-hours (OOH) primary care, depending on weekday and time of day.

There are three actors involved in emergency care in Germany and patients are free to choose one of them. First, hospitals participating in emergency care must ensure that they are able to provide emergency care for the life-threatening injured and sick [7]. Second, in the case of life-threatening, injured or sick persons and persons, who are expected to suffer serious damage to their health unless they receive immediate medical care, the emergency rescue service has to carry out the necessary medical measures at the place of the patient. Furthermore, the transportability of these persons must be determined and the treatment system suitable for further care must be carried out under professional supervision with the appropriate rescue equipment [8]. Third, OOH primary care is available for patients with non-urgent health problems who cannot wait until the regular consultation hours of their own GP [9]. All three sectors involved are free of charge (i.e. accessible without any co-payment) and obliged to treat any patient at any time.

The organization of the OOH primary care is quite variable: Different systems coexist like OOH primary care centres attached to a hospital, a set of participating practices or permanent OOH primary care without connection to a hospital [6]. Patients are forwarded to the OOH primary care centre in their region via the nationwide telephone number 116117. The number can be reached outside the regular opening hours of medical practices. No telephone contact is required to visit the OOH primary care centre. The opening hours of OOH primary care centres are not uniform, in smaller cities they are often only open one hour a day. If the OOH primary care centre is closed, the physician on duty can be reached directly by telephone via 116117. The patient then receives a telephone consultation or a home visit. Patients who are unable to visit the OOH primary care centre due to the severity of their condition may request a home visit. The obligation to participate in OOH primary care exists for all physicians, who work in outpatient care [10] but it is possible to look for a substitute (mostly GP or internist) [11]. In addition, there are a few OOH primary care centres especially for pediatric and ophthalmic patients that cover a larger catchment area.

In European countries different models of OOH primary care are established to provide effective patient care. The organization of OOH primary care differs not only between countries, but different models can also coexist within a country, as has been described for Germany, too [12–14]. The organization structures vary from individual practices and rota groups (several physicians within a practice look after their own and each other’s patients during OOH times) to larger general practitioner cooperatives (GPC). The dominant model in the Netherlands, Denmark und Switzerland is GPC, while the practice-based service model, where the individual physicians look after their own patients, is popular in Austria, Greece and Turkey [12]. In Norway strict gatekeeping is fundamental, so patients are not allowed to visit the ED directly, they must first contact a primary care center in case of emergency [15].

Research about OOH primary care and its quality has become more important in recent years and been conducted more often, but many aspects have not yet been sufficiently explored. In Germany, there are several studies on non-urgent visits to the EDs [16–19]. The few studies published so far on OOH primary care have only used administrative data [20, 21]. However, these data do not include information on the duration of complaints, waiting times on site, urgency of treatment from a medical point of view and the clinical pathways of patients.

The aim of this study was to determine patient characteristics, reasons for encounter (RFE) and its duration, diagnostics provided, medication prescribed, the necessity of hospital admission or hospital treatment as an outpatient, and the assessment of the urgency from the physicians’ point of view in an OOH primary care centre.

## Methods

### Design and setting

The data of this cross-sectional study were collected in a patient survey in an OOH primary care centre in Oldenburg, a city in northwestern Germany with a population of about 160,000 people within the federal state of Lower Saxony. It is also responsible for the surrounding rural areas, resulting in a total of 219,000 people covered. More than 100 physicians (most of them are GPs and internists) participate in the rotation system for the OOH primary care centre and its driving service. The OOH primary care centre is located next to a hospital near the city centre. It offers care between 7 pm to 10 pm on Mondays, Tuesdays and Thursdays and between 4 pm to 10 pm on Wednesdays and Fridays, when the GP offices are usually closed. On weekends and bank holidays it is open from 9 am to 10 pm. During the opening hours on weekdays, a physician and two practice nurses are present. On the weekends two physicians (one who carries out home visits) and additional practice nurses work there. Only limited diagnostics are possible in OOH, e.g. urine and blood rapid tests, ECG and ultrasound. For further diagnosis, the patient must be referred to the hospital.

In Oldenburg there are no other OOH primary care centres within a 20 km range. For children and adolescents there is an OOH service at the children’s clinic, which is provided by pediatricians. There is also an ophthalmological OOH service, which is carried out by ophthalmologists.

### Data collection

The cross-sectional study took place from 26 June until 23 July 2017. Data were collected via a two-sided questionnaire each day during the opening hours of the OOH primary care centre. All patients who had a contact with a physician at the OOH primary care centre during this period were contacted. Adults or children and adolescents, accompanied by their parents were included and no further exclusion criteria existed. Patients were approached by the research assistant in the waiting area. All participants had to give written informed consent. In the next step, the research assistant noted the date and time of arrival on the questionnaire and gave it to the patients. They filled out the first page about socio-demographic characteristics such as age, sex and region of residence. The type of health insurance was also asked for, since statutory and private health insurance differs in the reimbursement schemes for the medical services provided. Further questions were about having a regular GP, reasons for encounter (as free text), duration of complaints (today, 2–3 days, less or more than a week), and if an injury was the reason for coming. If the patients reported more than one reason for encounter (RFE), only the first symptom was assigned to an ICPC-2 (International Classification of Primary Care) category according to the official reason for encounter manual. ICPC-2 is a medical classification developed specifically for primary care in general medicine; instead of diagnoses, consultation reasons are coded [22]. Procedures such as dressing changes or injections as well as administrative questions were summarized in the category process codes.

The patients kept the questionnaire and handed it to the physicians. At the beginning of the consultation, the physicians noted the current time. Then they added information about diagnostic tests (blood tests and urinalysis, ultrasound, electrocardiogram and others as free text) and the following treatment like prescription of medication (analgesics/antipyretics, antibiotics and other as free text) and transfer to the ED (with or without admission to the hospital). At the end of the consultation, the physicians should assess whether the visit was necessary in OOH primary care centre or whether a GP could have done the treatment during the opening hours of the GP offices. The questionnaire was developed on the basis of previous experience of an OOH-GP and the research group. The questionnaire should be as short and precise as possible in order to minimize the burden on the participants. On a Saturday shortly before the start of the study, a pretest was carried out with all corresponding patients and the GP on duty. Few changes in wording were made. The data was collected anonymously. Questionnaires and declarations of consent were kept separately from each other.

### Statistical analysis

SPSS (IBM SPSS Statistics Version 23) and SAS for Windows version 9.4 were used for descriptive statistical analysis. The data was analyzed according to sex and age. The first group includes all minors while the other groups each incorporate intervals of ten years except the group between 18 and 29 years and older than 69 years. Analyses of patient characteristics, waiting time between arrival and first physician contact, first named reason for encounter, classification of complaints according to ICPC-2, further diagnostics, prescribed medication and hospital emergency admission were carried out. In addition, the physician’s subjective assessment of the urgency of treatment was evaluated. Missing data was not imputed.

## Results

In the four-week study period, 1098 patients visited the OOH primary care centre Oldenburg. Within this period, 188 h of treatment time were available, which corresponds to 5.8 patients per hour. A total of 29 different physicians worked in the OOH primary care centre during the study period, all of them were GPs or internists.

Of the 1098 patients a total of 892 gave their consent and participated in the study (response: 81.2%). Reasons for non-participation were not investigated. Nearly 40% of the participants were male; the mean age was 40.3 years (Table 1). Almost 70% of the patients lived in Oldenburg. 93.7% of the patients had a regular GP or a pediatrician. More than half (54.9%) of the patients fell in to the age group between 18 and 39 years, 28.9% into the age group between 40 and 59 years and 16.3% were at least 60 years old. Nearly 5% were children and adolescents; three quarters of them were older than 10 years. The average waiting time of the interviewed patients was 44.9 min. For 11.5% of respondents, the waiting time was more than 90 min. Most visits to the OOH primary care centre occurred on Saturday and Sunday (34.6% respectively 26.6% of all patients).

**Table 1:** Baseline characteristics of patients in an OOHC-centre (*n* = 892)

Characteristics	Proportion (95% confidence intervals [95% CI])
Sex (*n* = 890)	
Male	39.7% (36.4–43.0%)
Female	60.3% (57.0–63.6%)
Age Age in years (*n* = 877)	
Mean (95% CI); SD	40.3 (39.1–41.5%); SD: 18.5
Median [IQR]	36 [25–53]
Age groups in years	
0–17	4.8% (3.5–6.4%)
18–29	32.0% (28.9–35.2%)
30–39	18.1% (15.6–20.8%)
40–49	14.0% (11.8–16.5%)
50–59	14.9% (12.6–17.4%)
60–69	7.3% (5.6–9.2%)
70 +	9.0% (7.2–11.1%)
Place of residence (*n* = 887)	
City of Oldenburg	68.5% (65.4–71.6%)
Within catchment area of OOHC-centre	12.6% (10.5–15.0%)
Outside catchment area	18.8% (16.3–21.6%)
Medical insurance (*n* = 891)	
Social	88.3% (86.0–90.4%)
Private	10.8% (8.8–13.0%)
Other	0.7% (0.3–1.5%)
None	0.2% (0.0–0.8%)
Has regular GP/pediatrician (*n* = 886)	
Yes	93.7% (91.9–95.2%)
No	6.3% (4.8–8.1%)
Patients per weekday (n = 892)	
Monday (19–22)	5.3% (3.9–7.0%)
Tuesday (19–22)	4.5% (3.2–6.1%)
Wednesday (16–22)	11.1% (9.1–13.4%)
Thursday (19–22)	3.8% (2.7–5.3%)
Friday (16–22)	14.1% (11.9–16.6%)
Saturday (9–22)	34.6% (31.5–37.9%)
Sunday and holiday (9–22)	26.6% (23.7–29.6%)
Waiting time in minutes (*n* = 829)	
Mean (95% CI); SD	44.9 (42.5–47.3%); SD: 35.7
Median [IQR]	35 [15–70]
0–30 min	47.6% (44.2–51.1%)
31–60 min	20.5% (17.8–23.5%)
61–90 min	20.2% (17.5–23.1%)
91–120 min	9.0% (7.1–11.1%)
More than 120 min	2.7% (1.7–4.0%)

Patients between 18 and 29 years came more often during the week while older patients came more frequently on the weekend. From Monday to Friday, 38.8% of all patients between 18 and 29 years visited the OOH primary care centre compared to 27.6% during weekends.

### Reasons for encounter

**Fig. 1:**
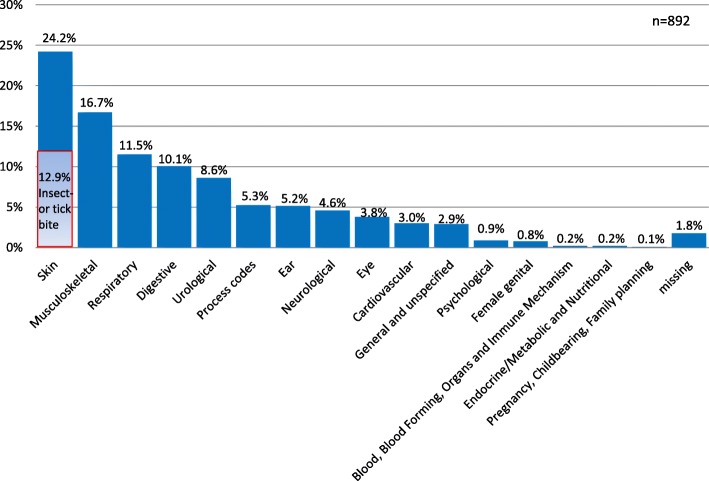
Reasons for encounter

In total, 866 (97.1%) patients mentioned a reason for encounter (Fig. 1). For 168 (19.4%) of them, two reasons and for 39 (4.5%) three reasons for encounter were reported. The first symptom was most frequently (24.2%) the ICPC-2 category “skin”, more than half of these patients had suffered insect or tick bites. A further 16.7% of the reasons were classified as “musculoskeletal”, just under the half of them with pain in back and neck. Infections of the upper respiratory tract (“respiratory”) were described by 11.5% of cases. Other frequent reasons for encounter were coded in “digestive” (10.1%), “urological” (8.6%) and “ear” (5.2%). The question of whether they come with an injury was answered by 15.9% of the interviewed patients with yes.

### Duration of symptoms

**Fig. 2:**
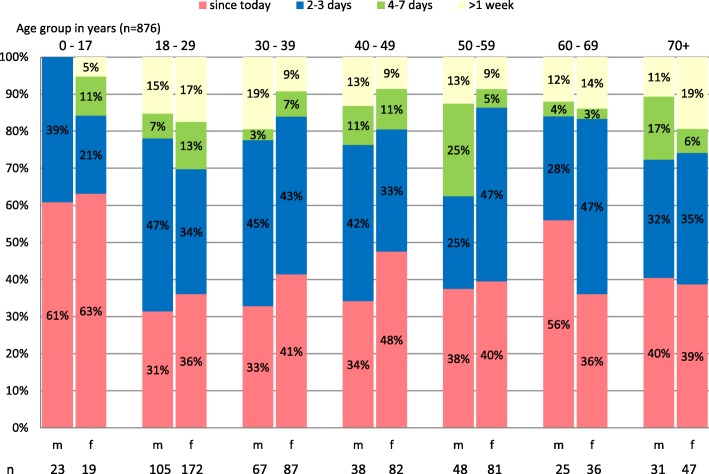
Duration of symptoms by age in years and sex

876 (98.2%) patients stated the duration of symptoms (Fig. 2). 61% of them had their symptoms for more than two days, including 13% for more than a week. For the remaining 39% of cases, the complaints first appeared on the day of consultation at the OOH primary care centre. A closer examination by age and sex shows that more than 60% of children and adolescents visit the OOH primary care centre with complaints that have existed “since today”. In the other age groups, there is no clear trend in the duration of symptoms. Between 31% (18–29 years old) and 56% (60–69 years old) of men visited the OOH primary care centre with symptoms “since today”, whereas this is the case for women between 36% (18–29 years old) and 48% (40–49 years old). In the 50–59 years age group, 38% of men reported that symptoms persisted for more than four days. Contrary to the other age groups, the duration of symptoms in men between 50 and 59 years of age lasted comparatively long (more than four days) before their visit.

### Medication

**Fig. 3:**
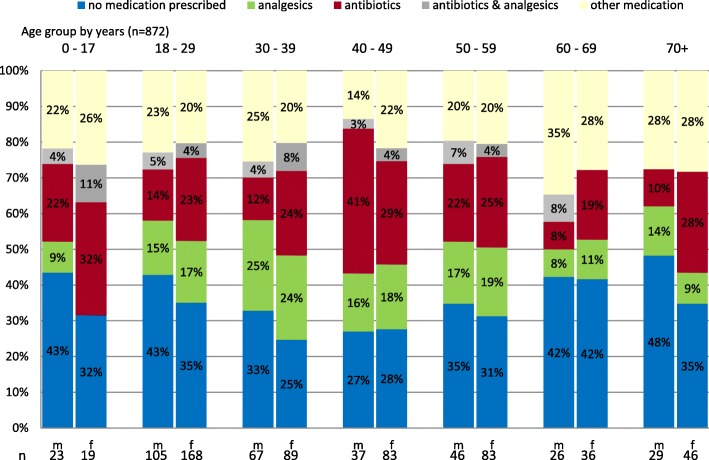
Medications prescribed by age in years and sex

Information on medication was given by the physicians for 872 (97.3%) patients (Fig. 3). In 34.5% of all cases no medication was prescribed. Antibiotics were most commonly prescribed in the age group of 40–49 years (women 29% vs. men 41%), and analgesics in the age group of 30–39 years (women 24% vs. men 25%). Almost one in two men and one in three women over the age of 70 years have not received any medication.

More than every fourth patient of the ICPC-2 categories “skin” and “urological” got a prescription for antibiotics (data not shown). Looking more closely at the category “skin”, 50% of cases with insect or tick bites received an antibiotic and another 12% for the prophylaxis after cat bites.

### Diagnostics tests

**Fig. 4:**
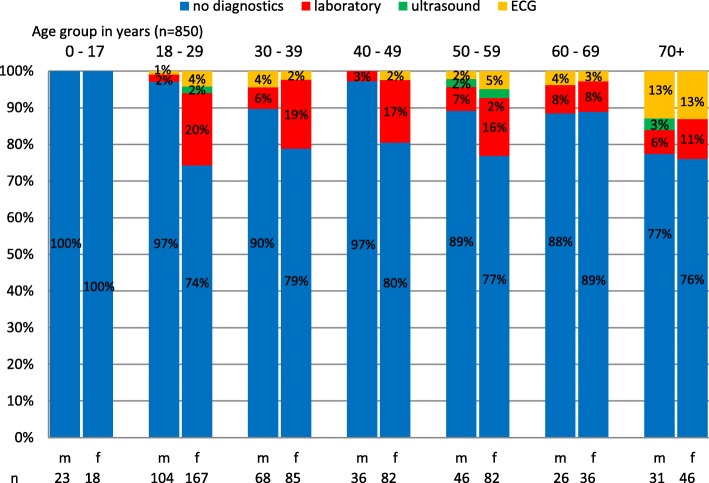
Clinical diagnostics

In 713 (83.9%) cases, anamnesis and physical examination were sufficient for the physician during the consultation (Fig. 4). Women received more than twice as many diagnostic tests as men (21% vs. 7.9%). Urinalysis and blood tests were the most frequently used diagnostic method (women 15.6% vs. men 4.2%). Nearly three quarters (73.9%) of these patients indicated urological complaints. In less than 1% of patients an ultrasound was performed. An electrocardiogram was performed on 3.7% of the patients.

### Urgency of consultation and hospitalization

**Fig. 5:**
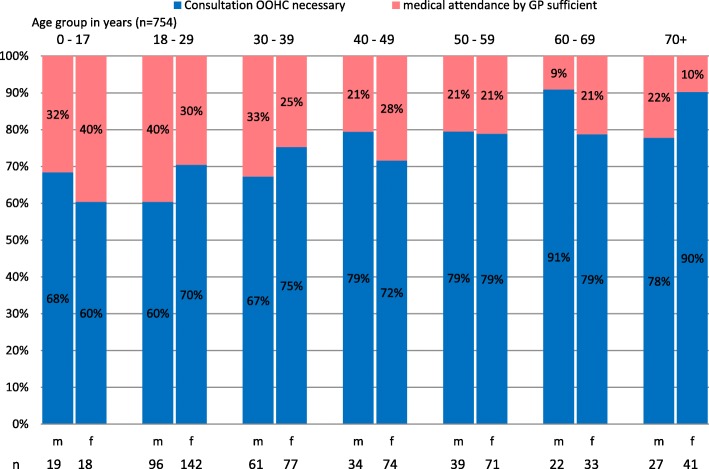
Urgency of consultation by age in years and sex

The subjective evaluation of the urgency of patient contacts was answered by the physicians for 768 (86.1%) cases (Fig. 5). According to the physician’s estimate, 24.5% of women and 28.9% of men could have been treated by their GP during regular opening hours. 32.7% of these cases reported symptoms in the ICPC category as “skin”, 13.9% of the cases related to “musculoskeletal” and 11.4% to “digestive”.

The number of necessary consultations increased with higher age. Patients younger than 30 years were the group with the highest share of patients which could have gone to a regular GP for consultation. While two-thirds of the consultations in this age group were rated as necessary, the proportion increased to up to 91% in the age of ≥60 years.

3.6% of patients were admitted to the hospital (data not shown). A further 4.5% were sent to the hospital for further clarification of the symptoms without admission. Hospital cases were mainly musculoskeletal (27.8%), digestive (16.7%) and circulatory (9.7%) disorders.

## Discussion

In our study, predominantly younger patients used the OOH primary care centre, more often on weekdays than on weekends. For more than half of the participants, symptoms lasted longer than two days. Nearly a quarter of the patients presented symptoms of the ICPC-2 classification “skin”. Only one in five patients received diagnostic tests and drugs were prescribed for two thirds. From the point of view of the attending physicians, a quarter of all patients could have been treated by a physician during regular consultation hours. In addition, nine out of ten patients remained in outpatient care.

### Findings and comparison to the literature

Younger people used the OOH primary care centre in Oldenburg more often than older people. In the literature, many studies have shown similar results in the proportion of younger patients receiving OOH primary care [16, 21, 23–25]. According to a study by Keizer et al., patients with non-urgent complaints were younger and often had a problem that lasted several days [26]. In addition, younger people often visited the OOH primary care out of convenience, while older people patients see some barriers to visiting OOH primary care. In particular, they avoid travelling late in the evening going to unknown places or making contact by phone Instead, they prefer to wait for a regular appointment with their GP [24]. Interestingly the proportion of younger patients up to 39 years was higher compared to the entire year (54.9% compared to 32.8% during 10/2016–09/2017).

More than a third of our study participants visited the OOH primary care centre on weekdays, indicating that the opening hours of GP offices are limited. In Germany, physicians’ practices are typically open until 6 p.m. on Mondays, Tuesdays and Thursdays and until around noon or 1 p.m. on Wednesdays and Fridays. Evening consultations or consultation hours on Saturdays are rarely offered. Therefore, some patients are unable to see their family doctor during office hours due to their working hours and are not able or willing to take time off from work [4, 27]. From the authors’ point of view, the increasing 24/7 availability could relate to patients no longer willing to wait for a regular appointment and prefer OOH treatment.

A quarter of all patients in our study reported symptoms from ICPC-2 category “skin” as reason for encounter. This means that the number of patients with skin problems is higher than in an international comparison. A study by Huibers et al. based on medical records from OOH primary care examined patient’s symptoms and diagnoses in European countries and showed that 15.5% of the RFE in Germany were categorized as ICPC-2 “skin”. Only in the Netherlands even more patients reported skin problems as a RFE (18.6%) [28]. Within the skin category, more than half of the study participants presented insect or tick bites and/or their consequences.

In addition to the ICPC-2 chapter “skin”, the ICPC-2 codes of the categories “general and unspecified”, “respiratory” and “musculoskeletal” were used most frequently in international comparisons [28]. Our study shows similar results. Every sixth patient presented with musculoskeletal pain and every tenth with infections of the upper respiratory tract.

More than 60% of patients have had symptoms for two days or more. If the symptoms have been present for some time, one might ask why the GP was not consulted earlier, during regular office hours and/or whether these patients could have waited until the next day. Due to the free choice of physicians in Germany, patients have free access to medical care at all times. This can lead to inappropriate visits to outpatient emergency care. An imperative telephone triage before visiting the OOH service could assess the urgency of the treatment and the need for immediate treatment. In some European countries this system has already been established in the OOH service [29–31].

83.9% of the patients did not receive any diagnostic measures. A study by Shipman et al. showed that the most important motives for contacting the OOH primary care centre were the need for advice, information and reassurance [32]. In another study, OOH service patients with non-urgent health problems mentioned concern for their own health and the need for medical information as reasons for visiting the OOH service [26]. The frequency of 16.1% patients receiving diagnostic tests is comparable with the results from other European countries with 5 to 20% [32–34]. Compared to the emergency department, comparatively few diagnostic examinations are carried out in an OOH primary care service. In contrast, the OOH primary care centres in the Netherlands and Norway are much better equipped [35, 36]. The different financing of country-specific health care systems might have an impact on the equipment of OOH services.

A quarter of all patients who presented themselves in the OOH primary care centre were judged as non-urgent by the physicians. From a medical perspective these patients could wait until the general practices are open or be managed by the patients themselves without further professional care [9]. International comparisons showed a wide range of these contacts likely to be unnecessary. In the Netherlands, nearly 42% of the patients in OOH primary care were classified as non-urgent (U4 + U5) [37] and in Denmark, 23.7% of all contacts to an OOH primary care centre were assessed as medically inappropriate [38]. In a Canadian study, 18% of the OOH primary care visits were rated as inappropriate by the GPs [39]. The assessment of urgency is a subjective perception of the patient and the physician. Not all physicians working in the study period would presumably come to the same conclusion. The results of Keizer et al. showed that two GPs, who judged the medical necessity on the basis of patient questionnaires, disagreed on 24% of the cases [26]. In Germany, there doesn’t exist a definition for the care of outpatient emergency patients in OOH primary care [40].

### Strengths and limitations

With 892 study participants, this study has a comparatively high number of cases, which allows differentiated analyses according to age and sex. Including information provided by the physicians in charge, we can understand better which cases are seen in an OOH primary care centre. In contrast, health insurance claims data do not allow insight into the duration of complaints or the need for treatment. Although the response was high, one fifth did not participate in the study. These patients might not have been able to answer the questions due to the acuity of their symptoms which would lower the proportion of non-urgent patients. Unfortunately, more detailed information was not recorded. The study was conducted in summer when insect and tick bites are more frequent and the number of respiratory infections is lower than in winter. A study covering a whole year would have probably changed the distribution of ICPC chapters. In addition, the study period was during the summer holidays, when some GP practices are closed and patients might be more willing to visit the OOH primary care centre than the substitute of the regular GP. The complaints described are based on the statements of the participants, which limits comparability with other studies. At the same time, the use of ICPC-2 has the advantage that a classification of complaints can be made and a dependency on diagnoses is eliminated. Our study does not rely on a more structured method like a triage system but on a subjective opinion so that the results depend on the personal views of the treating physician in each case. The transferability of the results to OOH primary care centres in other German regions could be restricted because of different organizational structures or other abilities of medical treatment. Regional characteristics like a certain population structure or local common diseases could impede the transferability likewise.

## Conclusion

This study provides a comprehensive insight into the work and medical care of an urban OOH primary care centre. The high proportion of younger patients with non-urgent complaints using the OOH primary care centre was remarkable in our study. In childhood, health competence and self-confidence could be strengthened in the family, at school and during regular GP visits in order to self-manage minor problems. Health care services should be made more transparent and comprehensible for the public. With this knowledge patients would be more aware of available health services, such as OOH primary care centres and EDs, and know when it is necessary to visit these emergency structures and when it is sufficient to go to a GP office. Further studies should investigate how many patients contacted the OOH primary care centre by telephone before a visit, how many patients were sent to the emergency department by the practice nurse due to the RFE without contact with the physician and whether they were referred back to the OOH primary care centre by the emergency department after triage if necessary. It would also be interesting to know whether the patient’s assessment of urgency is in line with the physicians’ assessment.

## Abbreviations

ED: Emergency department
GP: General practitioner
GPC: General practitioner cooperatives
OOH: Out-of-hours
